# Informal Emptying Business in Mandalay: Its Reasons and Financial Impacts

**DOI:** 10.1007/s00267-019-01228-w

**Published:** 2019-12-11

**Authors:** Wutyi Naing, Hidenori Harada, Shigeo Fujii, Chaw Su Su Hmwe

**Affiliations:** 1 Graduate School of Engineering, Department of Environmental Engineering, Kyoto University, Yoshida-honmachi, Sakyo, Kyoto 606-8501 Japan; 2 Graduate School of Global Environmental Studies, Kyoto University, Yoshida-honmachi, Sakyo, Kyoto 606-8501 Japan; 3 Department of Chemical Engineering, Mandalay Technological University, Patheingyi, Myanmar

**Keywords:** Fecal sludge management, Informal emptying business, Influential factor, Willingness-to-pay, Revenue loss

## Abstract

Globally, 2.8 billion people use on-site sanitation facilities, which need regular emptying of accumulated fecal sludge. Illegal dumping from informal emptying businesses, one of the major challenges in environmental management, is widely observed. Considering Mandalay, Myanmar, this study aimed to determine why informal emptying businesses are selected and estimate the lost revenue for a formal emptying service provider (FP) due to the informal businesses. We interviewed 400 households on their recognition and experiences regarding emptying services and willingness-to-pay for improved service. Revenue loss was estimated by comparing the present and theoretical maximum revenues. Results showed that 91.0% of households recognized FP only. Among 134 emptying-experienced households, 32.8%, 59.7%, and 4.5% chose FP with legal contact, FP with illegal contact, and informal service providers, respectively. The service fees from FP with illegal contact did not become revenue for FP; this was a major informal emptying business in the city. Differently from previous studies, the major illegal dumping was done by FP in this area. A great financial loss was estimated that FP lost 76.5% of the theoretical maximum revenue due to informal business. Logistic regression analysis indicated people’s intention to shorten the waiting time through illegal contact, even by paying a higher fee. As emptying services are typically required immediately after fecal sludge is over-accumulated, shorter waiting times and faster contact methods were the reasons why the informal business was selected. Less bureaucratic and more customer friendly system could reduce revenue loss, charge more, and increase profits.

## Introduction

Globally, 2.8 billion people rely on on-site sanitation such as septic tanks and pit latrines for their sanitation needs (WHO and UNICEF 2017). This is because centralized sanitation coverage is typically limited in urban areas owing to the associated costs. Worldwide, the number served by on-site sanitation is expected to grow to 5 billion by 2030 (Strande et al. 2014). On-site sanitation facilities accumulate fecal sludge and need to be emptied periodically to maintain their functionality. In low- and middle-income countries, 60% of the fecal sludge from on-site sanitation facilities is not safely managed; even if fecal sludge is safely collected, in many cases the untreated fecal sludge is improperly disposed of (Peal et al. 2014). Thus, improper fecal sludge management (i.e., FSM) is an environmental and public health hazard.

Informal emptying businesses, typically done by informal private emptying providers, are widely observed in many cities of developing countries: for instance, Dakar in Senegal (Mbéguéré et al. 2010), Jombang and Tegal in Indonesia (Tayler et al. 2013), Danang in Vietnam (Harada et al. 2015), and Phnom Penh and Siem Reap in Cambodia (Frenoux and Tsitsikalis 2015). The fecal sludge collected by informal businesses is typically disposed via open dumping or as illegal discharge to the sea, river, wasteland, and landfill sites (Chowdry and Kone 2012; Peal et al. 2014), and informal businesses may also lead to revenue losses and financial difficulties for formal service providers, resulting in further barriers to establishing sound fecal sludge emptying businesses (Taweesan et al. 2015). Therefore, a challenge in achieving sustainable FSM is to reduce informal businesses and to formalize the FSM process regardless of whether public or private businesses.

Several studies have considered the feasibility of fecal sludge emptying businesses. Frenoux and Tsitsikalis (2015) and Jenkins et al. (2015) confirmed crucial gaps between the potential emptying service demand and the capacity of the present service providers in Cambodia and Tanzania, respectively. Taweesan et al. (2015) and Murungi and van Dijk (2014) determined the factors influencing the emptying service quality, such as the number of households and collection trucks, truck capacity, and transportation distance. Halcrow et al. (2014) reported that the majority of a community in Bhutan had the willingness-to-pay for the existing emptying service. Mbéguéré et al. (2010) and Tayler et al. (2013) demonstrated that emptying services can be profitable. However, to the best of our knowledge, no study has focused on why and how informal emptying businesses are selected by people, and how formal emptying services regardless of whether public or private business should be improved to achieve sustainable FSM in developing countries.

Thus, the objectives of the present study were to determine why informal emptying businesses were selected, estimate its financial impacts on a formal provider and recommend measures for the improvement of emptying businesses in an urban area. Using Mandalay city, Myanmar, as a case study, we investigated the current situation in terms of emptying services with a focus on informal businesses and identified the impact of potential influential factors on the choice of emptying service types. Further, this study estimates the revenue loss of a formal emptying service provider (FP) due to informal businesses, and people’s willingness-to-pay for an improved emptying service.

## Materials and Methods

### Area of Study

Mandalay city is the second most populated city in Myanmar, with a population of 1.7 million (DOP 2015). The city is composed of five urban and two suburban townships. The study area encompassed the five urban townships, occupying 108.1 km^2^ (GAD 2017), with a population of 1.2 million (DOP 2015). The city is located at a hot and dry zone of Myanmar; the city has shallow underground water table (GAD 2017). The soil of the city is rich in clay followed by sand layer (MUSIP 2015). The open drainage system is used for the greywater management and there are neither sewer networks nor domestic wastewater treatment plant in the city.

### Emptying of Fecal Sludge in Mandalay City

The Water and Sanitation Department of Mandalay City Development Committee (i.e., MCDC-WSD) is the only FP of fecal sludge emptying services in the study area. Although private service providers are not formally permitted by the city, they were observed during our survey; this is a form of the informal emptying business in the city. There is a regulation that people have to visit the MCDC-WSD office to request the emptying services of the FP and pay the service fee at the office. In fact, people often do not visit MCDC-WSD but contact individual drivers by phone to request emptying services, negotiating the emptying fee by themselves. Although emptying services are provided by the FP’s drivers in this case, the emptying fee will not be paid to the MCDC-WSD office and does not constitute revenue earned by the MCDC-WSD; this is another form of the informal emptying business in the city. The collected fecal sludge by these informal emptying businesses was not received at the official disposal site, consequently leading illegal dumping.

According to our interview with MCDC-WSD officials, ten vacuum trucks were used for emptying septic tanks and pit latrines in 2017, all of which had a capacity of 4.5 m^3^ (MUSIP 2015). In comparison with the recommendation by Taweesan et al. (2015) of one truck per 1000 households, the number of trucks used by the MCDC was insufficient for the city. Moreover, the 4.5-m^3^ trucks require a road that is at least 3.0 m wide and therefore cannot enter the narrow roads that are scattered throughout the city, causing further challenges in terms of the emptying service coverage in the city. Although there are some small vacuum trucks assigned for drainage management in the Vehicle Department of the MCDC (i.e., MCDC-VD), the MCDC-WSD could not officially use these small trucks for any FSM purposes because of the bureaucratic system of the MCDC. The fecal sludge collection services offered by these small trucks are requested in an informal manner and the service fee does not constitute the revenue earned by either the MCDC-WSD or the MCDC-VD; this is also a form of the informal emptying business. As this is informal business, the emptied fecal sludge cannot be accepted at official dumping sites, causing illegal dumping of fecal sludge.

The city has two designated disposal ponds for fecal sludge, although only one was under operation as of 2017, located ~20 km away from the city center. Currently, there is no treatment mechanism inside the ponds and fecal sludge is simply dumped in the ponds. When the ponds are full, they are dredged and the sludge is dried beside the ponds. Illegal dumping on to the land inside the city by the MCDC-WSD vacuum trucks was observed. Similar to other cities discussed in Peal et al. (2014), the collected fecal sludge was unhygienically dumped into the environment.

### Interview Survey

Household interview surveys were conducted in the five urban townships of Mandalay city. The sample number of the survey was calculated so that the standard error was limited to ~5% at a 95% confidence interval. In total, 400 households were selected from the five urban townships. Each township was composed of 10–16 wards. In proportion to the population, 2–24 households were selected in each ward so that the sampled households were geographically scattered in each ward.

Questionnaires were prepared for obtaining demographic information, types of on-site sanitation facilities, fecal sludge management, recognition, and actual choices of emptying services, and willingness-to-pay for emptying services with different conditions (Table A.1 in the Supplementary Document). The willingness-to-pay index was estimated in an open-ended format. Respondents were asked which service they preferred and how much more money they could pay more for their preference: (1) service request options (phone call or office visit), and (2) methods used for emptying (mechanical or manual). The typical waiting time from service request to delivery was 1–3 weeks according to our interview with the MCDC-WSD. Then, respondents were asked how much money they could pay to shorten the waiting time from 21 to 14, 7, 3, 2, and 1 days.

### Revenue Calculation

This study assumed that the annual official revenue earned through emptying by FP (i.e., MCDC-WSD) was lower than the theoretical maximum annual revenue owing to informal emptying businesses. The theoretical maximum annual revenue was estimated as follows:

1 $$R_{\rm{max}} = C_f\,\times\,N_{\rm{OS}}\,\times\,F_{\rm{emptied}},$$

where *R*_max_ is the theoretical maximum annual revenue (USD/year), *C*_*f*_ is the official collection fee fixed by the FP (21.9 USD/time) (MUSIP 2015), *N*_OS_ is the number of on-site sanitation units in the city (213,104 units) (GAD 2017), *F*_emptied_ is the average frequency of emptying on-site sanitation facilities (time/unit/year). *F*_emptied_ was estimated as 0.079 time/unit/year using Eq. (2).

2 $$F_{\rm{emptied}} = \frac{{\mathop {\sum }\nolimits_i N_{\rm{emptied,i}}}}{{\mathop {\sum }\nolimits_i Y_{\rm{OS,i}}}},$$

where *N*_emptied,*i*_ is the number of times emptying was performed since the installation of on-site sanitation unit *i* based on our interview (time), *Y*_OS,*i*_ is the age of each on-site sanitation unit *i* based on our interview (year). For *N*_emptied_ and *Y*_OS,*i*_, we excluded on-site sanitation units with unknown ages and those older than 20 years to avoid considering extremely old units and because of the unreliable memory of respondents; still, 322 units were considered for this calculation, covering 80.5% of the total units investigated.

The present officially recorded annual revenue earned by FP was estimated as follows:

3 $$R_{\rm{present}} = C_f\,\times\,F_{\rm{recorded}}\,\times\,D_y,$$

where *R*_present_ is the present officially recorded annual revenue of the FP (USD/year); *F*_recorded_ is the daily average of the number of times emptying services were provided by the FP, which were officially recorded (15 times/day) (MUSIP 2015); and *D*_*y*_ is the number of working days of the FP in a year (264 days/year) (MUSIP 2015).

Finally, the annual revenue loss incurred by the FP was calculated using Eq. (4) as follows:

4 $$R_{\rm{loss}} \,=\, R_{\rm{max}} \,-\, R_{\rm{present}}$$

where *R*_loss_ is the estimated annual revenue loss incurred by the FP (USD/year).

### Statistical Analysis

The Kruskal–Wallis test was performed to identify significant differences in the potential influential factors among different emptying service conditions, followed by Dunn’s test of multiple comparisons. The logistic multivariable regression model was used to examine the impact of potential influential factors influencing the selection of formal or informal emptying services. The Friedman test was used to find significant differences in the willingness-to-pay index with respect to different emptying service conditions, while the pairwise Wilcoxon rank sum test was used to identify significant differences between household groups of different income levels. R version 3.4.4 (R Core Term (2018)) was used for the statistical analysis.

## Results and Discussion

### Toilet and Excreta Management

The information collected on the types of on-site sanitation facilities and experiences in terms of the emptying services from the 400 interviewed households is presented in the Supplementary Document (Table A.2). Briefly, the majority of the households (98.5%) used on-site sanitation facilities, while the remaining households (1.5%) discharged toilet wastes directly into water bodies. Septic systems were used by 84.7% of the interviewed households while 5.2%, 4.8%, and 3.8% of household using unlined pit latrine, lined pit latrine, and cesspool, respectively. In this study, septic tanks have two or more chambers with sealed bottom. Out of 337 septic tanks, 90% discharged effluent to open drainage and the remaining infiltrated into the ground. Cesspools have two or more chambers without sealing the bottom and without discharging of any effluent.

### Recognition of Emptying Service Providers

Out of the 400 interviewed households, 370 households (92.5%) recognized/knew at least one type of emptying service provider, while the remaining 30 households (7.5%) did not recognize any emptying service providers (Table 1). The FP providing mechanical emptying services was recognized by 92.2% of the households, while informal providers (IPs) offering mechanical emptying services unofficially using MCDC-VD small trucks (IPs-Me), and manual emptying services provided by private companies (IPs-Ma) were recognized by only 1.5%. IPs-Ma used buckets to remove fecal sludge from the storage tanks, mixed with sand and then discharged it to the environment nearby.

**Table 1:** Recognition of emptying service provider types by households

Recognized type of emptying service provider	Number of households
FP only	364 (91.0%)
IPs-Me only	1 (0.3%)
FP and IPs-Me	3 (0.7%)
FP and IPs-Ma	2 (0.5%)
No recognition	30 (7.5%)

*n* = 400. FP, IPs-Me, and IPs-Ma, respectively indicate the formal service provider, informal service provider offering mechanical emptying by using unofficial small trucks, and informal service providers offering manual emptying services

The results indicate that the majority recognized only the FP (364 out of 400) and there did not seem to be popular recognition of IPs. This contrasts with the survey data from a cross-sectional survey at 662 residential properties in 35 unplanned sub-wards in Dar es Salaam, Tanzania, where only 22.0% of the population was aware of FP (Jenkins et al. 2015). The dominant recognition of the FP observed in the present study could be advantageous for the study area to prevent improper emptying and dumping of fecal sludge.

In addition, because of advertisements and suggestions by their neighbors, 90 among 400 households had knowledge on using chemical additives as a measure to address the blockage problem of on-site sanitation facilities. The illegal direct discharge of fecal sludge in neighborhoods was recognized by 16 out of 400 households, although the present survey did not observe them.

### Actual Choice and Primary Contact for Requesting Emptying Services

Out of the 400 households, 134 (33.5%) had emptied their on-site sanitation facilities by the time we started our survey (i.e., experienced households). Four of the experienced households (one emptied 20 years ago, one emptied 3 years ago, and two did not answer when they emptied their facilities) did not disclose their choice of emptying services. A majority of the experienced households (124 households, 92.5%) chose the FP. This is in contrast to other cities in developing countries such as Jombang and Tegal in Indonesia (Tayler et al. 2013), Nonthaburi in Thailand (Harada et al. 2015), and Hanoi in Vietnam (Brandes et al. 2016), where a major proportion chose IPs. This primarily relates to the fact that most households in this study (91.0%) recognized only FP as emptying service providers.

Four and two households, respectively, used emptying services from IPs-Me and IPs-Ma, both of which are informal businesses. According to our interviews, IPs-Me was chosen for the areas accessible through narrow roads; however, IPs-Ma, which provides manual emptying services, was chosen owing to the low service fee and advantages over mechanical emptying in terms of breaking and removing strongly solidified sludge. These difficulties in the mechanical emptying services offered by FP were similar to those observed in a previous study in Kampala, Uganda (Murungi and van Dijk 2014).

Following the city regulation (Law No. 11/2014, MCDC), every household has to come to the MCDC-WSD office and pay fees to apply for emptying services. Apart from visits to the office, other methods of emptying service requests resulted in informal businesses. Illegal ways for requesting emptying services were classified into three groups according to their primary contact method: phone call to the office (21.0%), phone calls to FP drivers (30.6%) and asking a friend (12.9%) (Table 2). Here, providing emptying services upon receiving requests via phone was considered illegal because such emptying service requests were not officially recorded by the MCDC-WSD and the service fee was not paid to the office but to the drivers. Regarding the request for emptying services through friends, according to our interview, the household paid the emptying service fee not to the MCDC-WSD office but directly to the FP drivers; thus, this also constitutes informal business. It is noteworthy that the majority of households contacted FP in an illegal manner (64.5% of experienced households).

**Table 2:** Different primary contact methods to formal service providers

	Knowledge of the regulation		Total
	With knowledge no.	Without knowledge no.	
Legal contact (office visit*)*	38	6	44 (35.5%)
Illegal contact	34	46	80 (64.5%)
Phone to the office	13	13	26 (21.0%)
Phone to a driver	15	23	38 (30.6%)
Asking a friend	6	10	16 (12.9%)
Total	72 (58%)	55 (42%)	124 (100%)

Office visit is the only legal contact method, and other contact methods are illegal in the study area. Samples constitute experienced households who chose the formal service provider (FP)

**Fig. 1:**
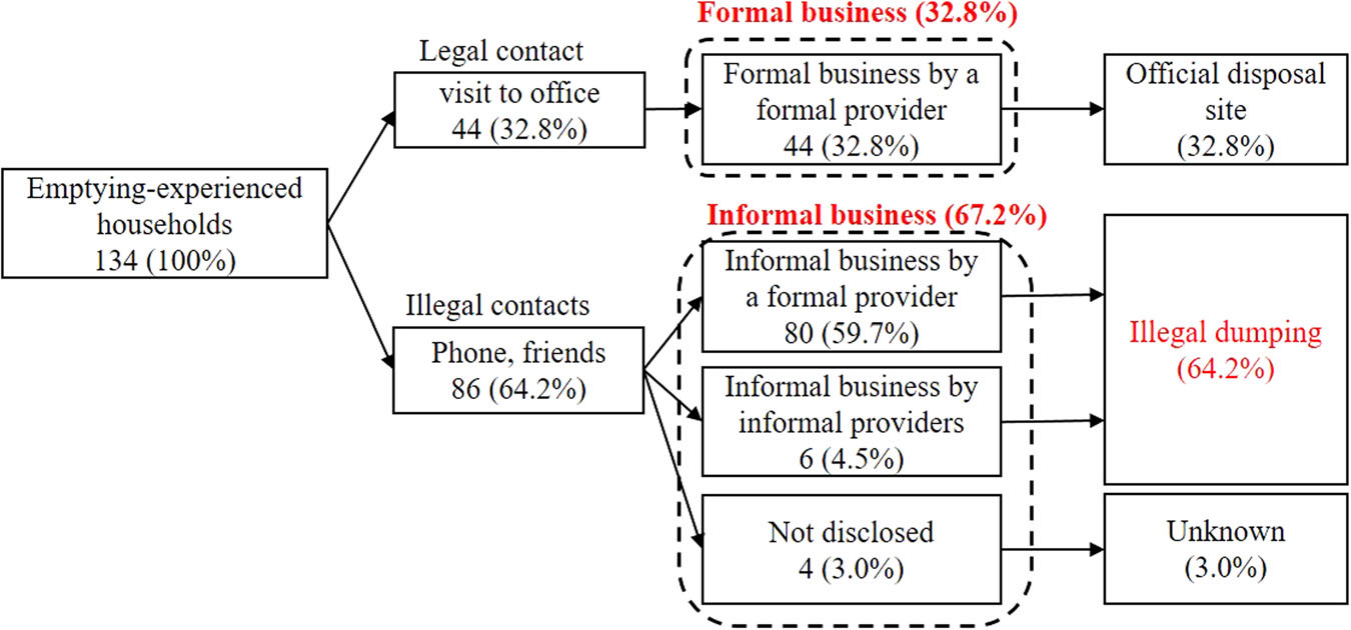
Choice of fecal sludge emptying services. Data obtained from emptying-experienced household (*n* = 134). The number indicate household numbers and their proportions

Furthermore, although 72 households (58.0%) recognized this regulation, 34 still contacted the FP in an illegal manner. The fees earned by providing emptying services through these illegal contacts did not become the FP’s official revenue. These results indicate that although the service was provided by FP, a significant proportion of the emptying services was provided through illegal contacts, resulting in informal business. Whereas previous studies revealed that private informal businesses were big challenges along with illegal dumping, this study confirmed the illegal dumping from public sector is a major challenge. Formalization of emptying businesses is important to reduce illegal dumping regardless of whether public or private emptying service provider. Including the IP-Me, IP-Ma, and FP services availed through illegal contacts, the informal emptying business accounted for 67.2% of the total emptying services among the interviewed households (Fig. 1), leading to illegal dumping of fecal sludge.

### Factors Influencing the Choice of Emptying Services

**Fig. 2:**
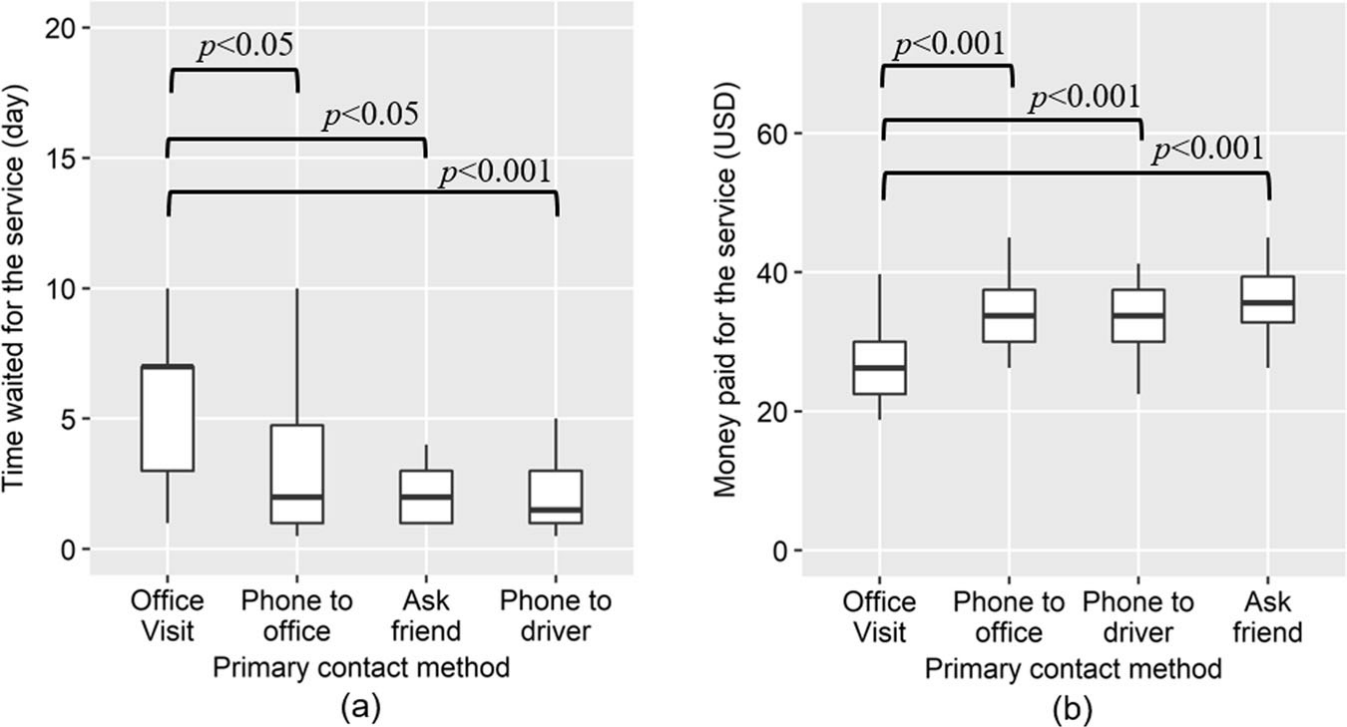
Waiting time and service fee for the formal emptying service provider based on different primary contact methods. **a** Waiting time in different contact methods (*n* = 103). **b** Service fee associated with different contact methods (*n* = 103). Samples of analyzed households opting for emptying services provided by the formal service provider (FP) within the last 3 years. The statistical difference was tested using the Kruskal–Wallis test followed by Dunn’s test of multiple comparisons. An office visit is the only legal contact method, and the other methods are illegal in the study area. Boxplots indicate median and interquartile ranges (IQRs) with whiskers extending up to 1.5 × IQR from the median

The method of contacting the service providers was an important factor for distinguishing between formal and informal emptying businesses as mentioned in the previous section. The factor influencing the selection of legal and illegal contact methods were analyzed for the households that availed the services of FP within the last 3 years considering five potentially influential factors: income level, toilet type, education level, waiting time from service request to service delivery, and emptying service fee paid by a household. Households that emptied their facilities within the last 3 years were chosen to collect reliable information on the service fee and waiting time. As observed in Table 3, the paid service fee (*p* < 0.001, negative estimate) and waiting time (*p* = 0.048, positive estimate) significantly influence the choice of the contact methods. Considering these results and the *z* scores in the regression model, illegal contact was dominantly associated with a higher service fee and shorter waiting time, indicating that people chose illegal contact methods for a shorter waiting time but paid higher service fees. This was supported by the comparison of the waiting time and paid service fee among four different methods for contacting emptying service providers, shown in Fig. 2. Requesting emptying services through legal contact methods (i.e., office visit) required a significantly longer waiting time but involved a lower service fee than the other three illegal contact methods.

**Table 3:** Logistic regression table for factors influencing the choice of contact method

Coefficients	Estimate	Std. Error	*z* value	*p*	OR (95% CI)
(Intercept)	4.63	1.99	2.32	0.020*	–
Income level (1–5)	−0.01	0.19	−0.04	0.968	1.10 (0.82, 1.48)
Toilet type (0, 1)	0.43	0.80	0.53	0.595	1.58 (0.50, 5.02)
Education level (1–5)	0.04	2.64	0.16	0.870	0.99 (0.69, 1.42)
Waiting time (day)	0.15	0.08	1.97	0.048*	1.27 (1.11, 1.46)
Paid service fee (USD)	−0.20	0.05	−3.62	<0.001***	0.82 (0.73, 0.89)

Samples analyzed are households that availed emptying services offered by the formal service provider (FP) within the last 3 years (*n* = 103). Choices of contact methods were classified as 1: legal (visit office) and 0: illegal. Monthly income level was classified as 1: <200 USD, 2: 200–300 USD, 3: 300–400 USD, 4: 400–500 USD, and 5: >500 USD. Education level was classified into 1: less than primary school, 2: primary school, 3: secondary, 4: diploma, 5: graduate, and 6: more than one degree and other. Toilet type was classified into 1: septic tank and 0: others (cesspool, pit latrine, no facility, direct connection to water body, and lined pit latrine)

*p < 0.05; ***p < 0.001

Thus, it is evident that many people intend to shorten the waiting time by contacting FP in an illegal manner, even when they recognize the regulation for requesting emptying services and need to pay a higher fee. This tendency may be explained by their urgent need for emptying services. Similar to the results of previous studies in cities such as Hanoi, Vietnam (Harada et al. 2008), Kampala, Uganda (Etajak 2010; Kulabako et al. 2010), and Thimphu, Bhutan (Halcrow et al. 2014), people in the present study area requested emptying services only when their on-site sanitation facilities had serious troubles such as blockage and overflow. This indicates the immediate need for service delivery and a faster way to request for the service (e.g., contact through phone call). This suggests that if FP made their procedures less bureaucratic, and more customer friendly, they could also charge more and increase their profit. Further, as other cities in developing countries also require immediate emptying after fecal sludge is over-accumulated and households face serious troubles in terms of on-site sanitation, these improvements in emptying businesses may expand opportunities for formal emptying not only in Mandalay but also in other cities, leading to improved financial conditions of FP in developing countries.

### Emptying Business of the Formal Service Provider

The theoretical maximum and present revenues of the emptying business were estimated for the whole city using Eqs. (1)–(4). The present revenue of FP from their emptying service was estimated to be 86,700 USD per year, which is 23.5% of the theoretical maximum revenue, 368,700 USD per year. This indicates that FP potentially lost 76.5% of the theoretical maximum revenue owing to informal business, equivalent to 282,000 USD per year. As mentioned above, the total share of informal business was estimated at 67.2% of the emptying services in the city. Still, these proportions coincide to some degree with each other although a 9.3% gap exists between these two proportions. The gap can be attributed to the following reasons. First, both proportions were estimated based on the data of emptying experience reports provided by respondents, which potentially contain errors owing to their memory. Second, our data contained sampling error: frequency of emptying on-site sanitation facilities (0.079 ± 0.024 unit/time/year) and the informal business proportion (0.672 ± 0.041). Third, the 67.2% share was calculated by using the answers provided by respondents regarding the type of emptying work. Some respondents who used illegal emptying services might not provide honest responses, potentially resulting in a lower illegal proportion being calculated.

This lost revenue possibly flows into informal emptying businesses in the city, including the emptying services offered by FP through illegal contacts, IPs-Me, and IPs-Ma. Previous studies have reported that providing emptying services could be a profitable business, for instance in Jombang, Indonesia (Tayler et al. 2013), and in Ningo–Prampram district, Ghana (Nimoh et al. 2014). In the study area, financial burden is still the biggest challenge. Considering the significant amount of lost revenue, formalization of informal emptying business could substantially increase the revenue of FP, leading to the improvement of FSM in the city.

### Willingness-to-pay for Emptying Services in Different Conditions

The above results indicated that the main reason for choosing the more expensive informal emptying services was to shorten the waiting time by contacting the provider in a fast manner. This section discusses the willingness of people to pay for emptying services considering different conditions factors (waiting time, and contact manner).

#### Waiting time

**Fig. 3:**
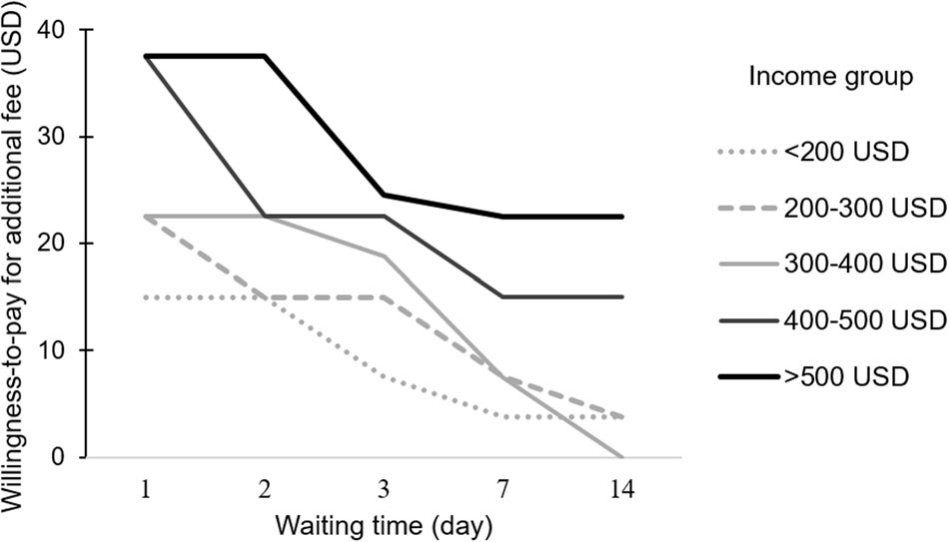
Comparison of median of willingness-to-pay index with the waiting time reduced from 3 weeks in each income level (*n* = 400). Willingness-to-pay index for each income level was significantly different according to the change in waiting time (*p* < 0.001; Friedman test), and among income levels (*p* < 0.001; Kruskal–Wallis test). Results of pairwise Wilcoxon rank sum tests are summarized in Tables A.3 and A.4 in the Supplementary Document.)

For all the respondents including experienced and inexperienced households (*n* = 400), fig. 3 presents the willingness-to-pay index for five income groups to reduce the waiting time fixed by FP from 3 weeks (maximum waiting time from our survey) to five different waiting times. The willingness-to-pay index of all income groups significantly increased on reducing the waiting time (*p* < 0.001; Friedman test). Significant differences in the willingness-to-pay index were confirmed for different income levels (*p* < 0.001, Kruskal–Wallis test). Further, significant differences were observed in all of pairs of waiting times and all of pairs of income groups except the income pairs among the lowest (<200 USD/month), lower-middle (200–300 USD/month), and middle (300–400 USD/month) groups (Tables A.3 and A.4 in Supplementary Document). The willingness-to-pay index of the 400–500 USD/month and >500 USD/month groups was significantly higher than the other lower income groups (Table A.4 in Supplementary Document). Results indicated that regardless of their income levels, people are willing to pay for shorter waiting times; particularly, a higher willingness-to-pay index to reduce waiting time was observed for people with higher income levels.

#### Contact method

**Fig. 4:**
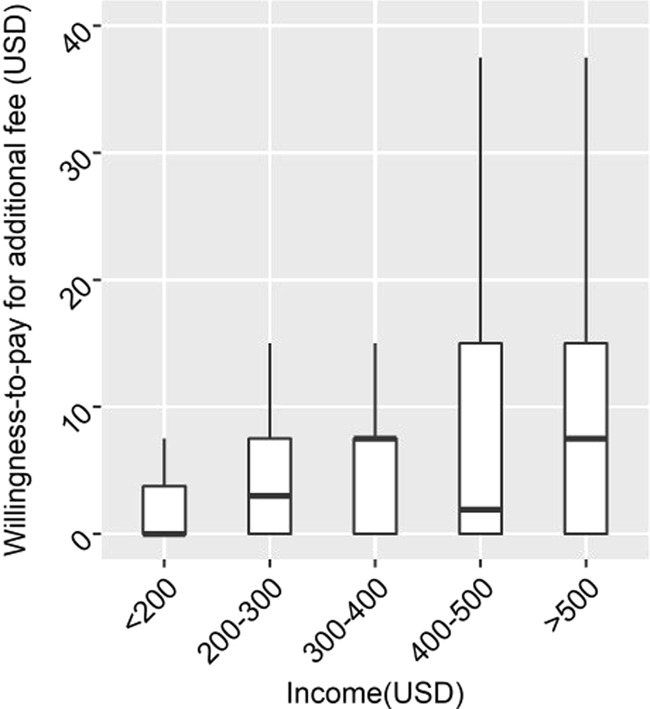
Willingness-to-pay index with respect to additional fees for requesting emptying services through telephonic contact rather than visiting office (*n* = 400). Statistical difference of each pair was tested via pairwise Wilcoxon rank sum tests and the results are summarized in Tables A.5 in the Supplementary Document. Boxplots indicate the median and IQRs with whiskers extend up to 1.5 × IQR (IQR from the median)

The willingness-to-pay index regarding the additional service fee to request emptying services through a phone call rather than visiting the office is presented in Fig. 4. Out of the 400 households interviewed, 232 households (58.0%) showed the willingness-to-pay. The median of the willingness-to-pay index ranged between 7 USD and 14 USD for the five income groups; the higher the income, the higher was the additional fee they were willing to pay. Significant differences in the willingness-to-pay index was observed among income groups (*p* < 0.001, Kruskal–Wallis test), and the high-income group (>500 USD/month) showed a significantly higher willingness-to-pay index than the low (<200 USD/month) and lower-middle-income groups (200–300 USD/month) (pairwise Wilcoxon Rank Sum Tests), as summarized in Table A.5 in the Supplementary Document. These results correspond to those discussed above that even if the service fee was higher, the emptying services by FP through illegal contact methods was in fact widely used in the city (Fig. 2b). Jenkins et al. (2015) suggested that the easy availability of emptying services was one of the factors affecting the choice of services in Dar Es Salaam, Tanzania. In line with Jenkins et al. (2015), our results further indicate that the choice of contacting the service provider via phone could significantly increase the value of emptying services and that people would be willing to pay for such services.

## Conclusions

As a case study in Mandalay, Myanmar, we analyzed fecal sludge emptying services for on-site sanitation facilities with a focus on the choice of informal emptying services. In contrast to other cities, most of the emptying-experienced households (92.5%) in the study area used FP, which primarily related to the fact that most households in the city (91.0%) recognized only FP. However, the majority of the experienced households (64.5%) requested emptying services from FP in an illegal manner; this was a major form of informal emptying businesses in the city. A great financial loss was estimated that FP lost 76.5% of the theoretical maximum revenue owing to informal emptying businesses. Logistic regression analysis indicated that the waiting time until emptying service delivery and the service fee were significant parameters influencing the method used to contact FP. People focused on shortening the waiting time even though they chose illegal methods to contact and paid higher fees. This is supported by the higher willingness-to-pay index for shortening the waiting time and quickly contacting the provider.

In conclusion, informal emptying business financially burdened formal emptying business greatly. A shorter waiting time and fast way of contacting the service providers are the main reasons for people to select informal emptying services. Less bureaucratic and more customer friendly procedure, could reduce the revenue losses, charge more for their services, and increase their profit. Similarly in Mandalay, many cities in developing countries also require immediate emptying after fecal sludge is over-accumulated and households face serious troubles regarding on-site sanitation facilities. Immediate service delivery and fast methods of contact are, therefore, suggested as key measures for formalizing the informal emptying business, regardless of whether public or private business to avoid illegal dumping not only in the study area but also in other cities. Although the present paper did not suggest a model for the emptying business in the city, the insights regarding the preferences of most households and their willingness-to-pay for emptying services could contribute toward improving emptying services in developing countries, leading to the better FSM.

## Supplementary Information

Supplementary Appendix

## References

[CR1] Brandes K, Schoebitz L, Nguyen V-A, Linda S (2016) SFD promotion initiative—Hanoi Vietnam—final report. Eawag (the Swiss Federal Institute of Aquatic Science and Technology), Duebendort

[CR2] Chowdry S, Kone D (2012) Business analysis of fecal sludge management: emptying and transportation services in Africa and Asia. The Bill & Melinda Gates Foundation, Seattle

[CR3] DOP (Department of Population) (2015) The republic of the union of Myanmar. The 2014 Myanmar Population and Housing Census report. Vol 3–1. Ministry of Immigration and Population, Naypyitaw

[CR4] Etajak S (2010) The financing of sanitary facilities in urban slums in Kampala. A case study of Bwaise slum. MSc thesis, Makerere University, Kampala

[CR5] Frenoux C, Tsitsikalis A (2015). Domestic private fecal sludge emptying services in Cambodia: between market efficiency and regulation needs for sustainable management. J Water Sanit Hyg Dev.

[CR6] GAD (General Administration Department) (2017) Regional data report. GAD, Mandalay

[CR7] Halcrow G, Yetsho T, Nguyen N, Tshering G (2014). Developing behaviour change communication for improving faecal sludge management in Bhutan. J Water Sanit Hyg Dev.

[CR8] Harada H, Dong NT, Matsui S (2008). A measure for provisional-and-urgent sanitary improvement in developing countries: septic-tank performance improvement. Water Sci Technol.

[CR9] Harada H, Schoebitz L, Linda S (2015) SFD promotion initiative Nonthaburi Thailand—final report. Eawag (the Swiss Federal Institute of Aquatic Science and Technology), Duebendort

[CR10] Harada H Schoebitz L, Strande, L (2015) SFD promotion initiative—Danang (Report). Eawag (the Swiss Federal Institute of Aquatic Science and Technology), Duebendort

[CR11] Jenkins MW, Cumming O, Cairncross S (2015). Pit latrine emptying behavior and demand for sanitation services in Dar Es Salaam, Tanzania. Int J Environ Res Public Health.

[CR12] Kulabako N, Nalubega M, Wozei E, Thunvik R (2010). Environmental health practices, constraints and possible interventions in peri-urban settlements in developing countries—a review of Kampala, Uganda. Int J Environ Health Res.

[CR13] Mbéguéré M, Gning JB, Dodane PH, Koné D (2010). Socio-economic profile and profitability of faecal sludge emptying companies. Resour Conserv Recycl.

[CR14] Murungi C, van Dijk MP (2014). Emptying, transportation and disposal of feacal sludge in informal settlements of Kampala Uganda: the economics of sanitation. Habitat Int.

[CR15] MUSIP (Mandalay Urban Services Improvement Project) (2015) TA-8472 MYA: preparing Mandalay urban services improvement project final report, Vol 5. Mandalay City Development Committee (MCDC), Mandalay

[CR16] Nimoh F, Poku K, Ohene-Yankyera K, Konradsen F, Abaidoo RC (2014). Constraints and motivations to sanitation business in peri-urban communities in Ghana. J Water Sanit Hyg Dev.

[CR17] Peal A, Evans B, Blackett I, Hawkins P, Heymans C (2014). Fecal sludge management: a comparative analysis of 12 cities. J Water Sanit Hyg De.

[CR18] R Core Team (2018) R: a language and environment for statistical computing. R Foundation for Statistical Computing, Vienna, Austria. https://www.R-project.org/

[CR19] Strande L, Ronteltap M, Brdjanovic D (2014) Faecal sludge management. Vol 9781780404. IWA Publishing, London

[CR20] Taweesan A, Koottatep T, Dongo K (2015). Factors influencing the performance of fecal sludge management services: case study in Thailand municipalities. Environ Dev Sustain.

[CR21] Taweesan A, Koottatep T, Polprasert C (2015). Effective faecal sludge management measures for onsite sanitation systems. J Water Sanit Hyg Dev.

[CR22] Tayler K, Siregar R, Darmawan B, Blackett I, Giltner S (2013). Development of urban septage management models in Indonesia. Waterlines.

[CR23] WHO and UNICEF (2017) Progress on drinking water, sanitation and hygiene: 2017 update and SDG baselines. World Health Organization (WHO) and the United Nations Children’s Fund (UNICEF), Geneva. Licence: CC BY-NC-SA 3.0 IGO

