# Supplementary material for: Informal Emptying Business in Mandalay: Its Reasons and Financial Impacts
Source: Environ Manage. 2019 Dec 11;65(1):122–30. doi: 10.1007/s00267-019-01228-w (PMC6960235; doi:10.1007/s00267-019-01228-w)
Supplement: Supplementary file 1 — Supplementary Appendix [file 267_2019_1228_MOESM1_ESM.docx]

**Informal emptying business in Mandalay: its reason and financial impacts**

Wutyi Naing, Hidenori Harada^*^, Shigeo Fujii, Chaw Su Su Hmwe

Journal Name: Environmental Management

Corresponding to: Hidenori Harada, Kyoto University, Japan

E-mail: [harada.hidenori.8v@kyoto-u.ac.jp](mailto:harada.hidenori.8v@kyoto-u.ac.jp)

**APPENDIX**

**Table A.1. Summary of information collected from the questionnaire survey**

| Questionnaire content | Detail information |
| --- | --- |
| Demographic information | Income  Education  Household size |
| Onsite sanitation facilities | Type of onsite sanitation facilities  Age of onsite sanitation facilities |
| Septage management | Have been emptied/never emptied  Emptied frequency  Latest emptied time  Knowledge for emptying service  Choice of emptying service  Reasons of choice  Contacted way for the emptying service  Service fee paid for the emptying service  Waited time for the emptying service |
| Preference and willingness-to-pay for emptying service | Preferred types of service (mechanical/manual)  Willingness-to-pay additional fee for mechanical service over manual service  Preferred type of contacted ways (phone/go to office)  Willingness-to-pay additional fee for fast contact way over visiting to office  Willingness-to-pay additional fee for shorter waiting time until the arrival of emptying services |

**Table A.2. Types of onsite sanitation facilities and experiences in regard to emptying services**

| Onsite sanitation facility | *n* | % | Type of emptying service | | | Never emptied |
| --- | --- | --- | --- | --- | --- | --- |
|  |  |  | FP | IPs-Me | IPs-Ma |  |
| Private toilet |  |  |  |  |  |  |
| Septic tank | 337 | 84.2% | 108 | 4 | 2 | 219 |
| Unlined pit latrine | 21 | 5.2% | 2 | - | - | 19 |
| Lined pit latrine | 19 | 4.8% | 10 | - | - | 9 |
| Cesspool | 15 | 3.8% | 4 | - | - | 11 |
| Toilet directly connected to water body | 6 | 1.5% | - | - | - | 6 |
| Shared/public toilet |  |  |  |  |  |  |
| Septic tank | 2 | 0.5% | - | - | - | 2 |
| Total | 400 | 100% | 124 | 4 | 2 | 266 |

Note: Four households with septic tanks who had emptied did not disclose the service type they chose. FP, IPs-Me and IPs-Ma, respectively, indicates the formal service provider, the informal service provider that provide mechanical emptying by using unofficial small trucks, and the informal service providers that provide manual emptying.

**Table A.3. Results of statistical difference tests of willingness-to-pay more money to reduce waiting time from three weeks**

| Waiting time  (*p* value) | 14 days | 7days | 3days | 2days |
| --- | --- | --- | --- | --- |
| 7days | *p*<0.001 |  |  |  |
| 3days | *p*<0.001 | *p*<0.001(***) |  |  |
| 2days | *p*<0.001 | *p*<0.001 (***) | *p*<0.001(***) |  |
| 1day | *p*<0.001 (***) | *p*<0.001 (***) | *p*<0.001(***) | *p*<0.001(***) |

Note: *n*=400. Statistical difference was tested by the Pairwise Wilcoxon rank sum test.

**Table A.4. Results of statistical difference tests of willingness-to-pay more money within different income levels to reduce waiting time from three weeks**

| Income group  (*p* value) | <200$ | 200-300 | 300-400 | 400-500 |
| --- | --- | --- | --- | --- |
| 200-300 | *p=*0.066 |  |  |  |
| 300-400 | *p*<0.01 (**) | *p*=0.153 |  |  |
| 400-500 | *p*<0.001(***) | *p*<0.001 (***) | *p*<0.01 (**) |  |
| >500 | *p*<0.001 (***) | *p*<0.001 (***) | *p*<0.001(***) | *p*<0.05(*) |

Note: *n*=400. Statistical difference was tested by the Pairwise Wilcoxon rank sum test.

**Table A.5. Results of statistical difference tests of willingness-to-pay more money for phone contact emptying service by different income levels**

| Income group  (*p* value) | <200$ | 200-300 | 300-400 | 400-500 |
| --- | --- | --- | --- | --- |
| 200-300 | *p*=0.222 |  |  |  |
| 300-400 | *p*<0.01 (**) | *p*=0.222 |  |  |
| 400-500 | *p*=0.222 | *p*=0.860 | *p*=0.860 |  |
| >500 | *p*<0.001 (***) | *p*<0.01 (**) | *p*=0.222 | *p*=0.174 |

Note: *n*=400. Statistical difference was tested by the Pairwise Wilcoxon rank sum test.


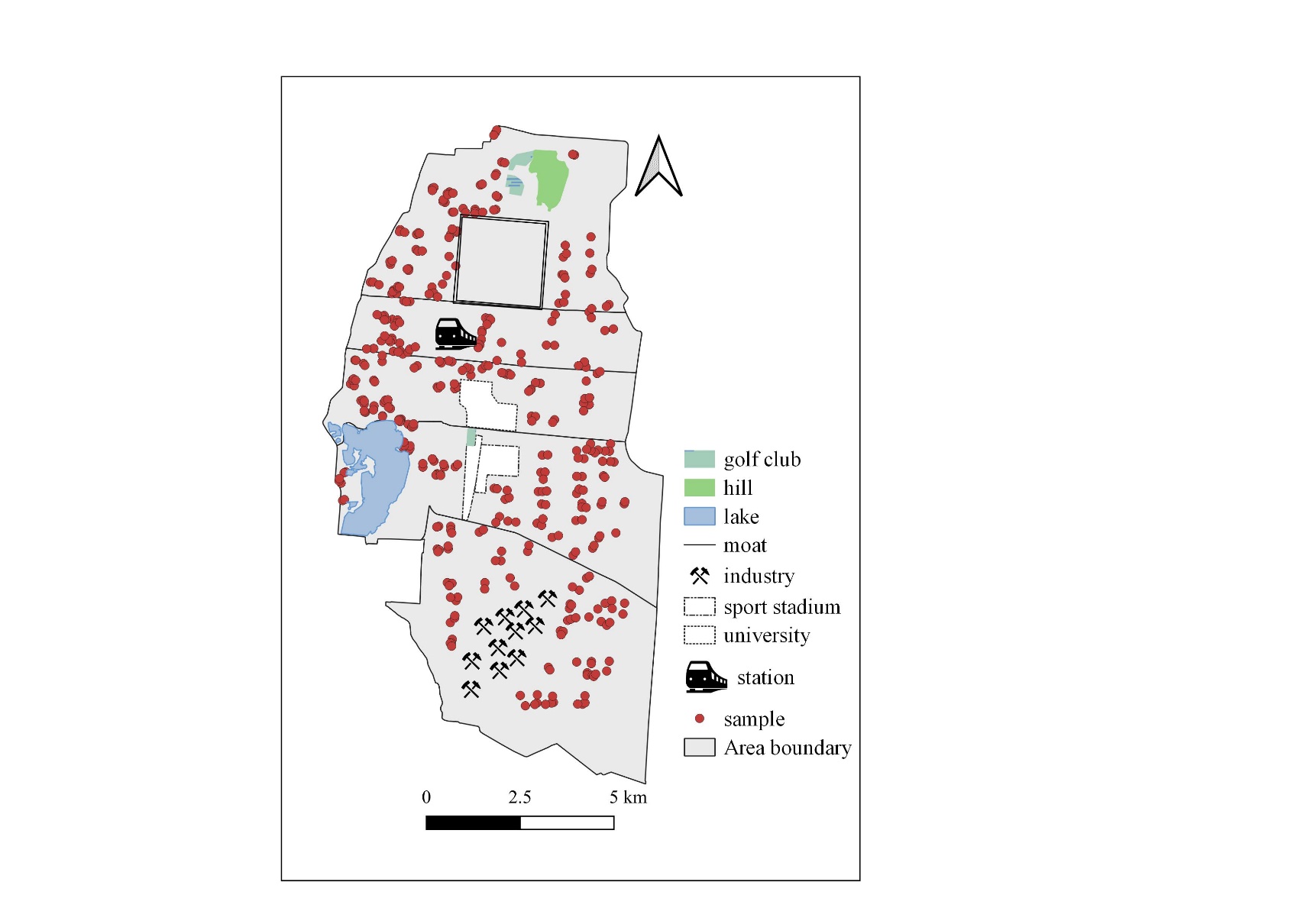


Figure A.1 Map of Sample distribution. Area boundary was the administrative boundary of five unban townships (Aung Myae Tharzan, Chan Aye Tharzan, Mahar Aung Myae, Chan Mya Tharzi and Pyi Gyi Takon) in Mandalay city, Myanmar.
